# A rare case report: co-occurrence of two types of lung cancer with hamartoma and pulmonary tuberculosis

**DOI:** 10.3389/fonc.2023.1264871

**Published:** 2023-10-04

**Authors:** Jianxiong Kang, Mu Wang, Peiyan Hua, Bin Wang

**Affiliations:** Department of Thoracic of the Second Hospital of Jilin University, Changchun, Jilin, China

**Keywords:** lung adenocarcinoma, lung squamous cell carcinoma, pulmonary tuberculosis, pulmonary hamartoma, case report

## Abstract

With the widespread use of low-dose chest Computed Tomography (CT), lung nodules are being increasingly detected. Common pulmonary conditions such as lung adenocarcinoma, lung squamous cell carcinoma, and tuberculosis are typically diagnosable through imaging examinations. Nevertheless, when multiple types of lung cancer are combined with other benign tumors, how can an accurate diagnosis be made? In this report, we present a rare case of a patient with the simultaneous occurrence of lung adenocarcinoma, lung squamous cell carcinoma, pulmonary tuberculosis, and pulmonary hamartoma, which has not been previously reported. This patient underwent surgical intervention in the Department of Thoracic Surgery at the Second Hospital of Jilin University and has now fully recovered and been discharged. The patient’s preoperative positron emission tomography-CT(PET-CT)results did not align with the postoperative pathological diagnosis. The imaging findings were atypical, and the pathological diagnosis was exceptionally rare. We share this case report to contribute to the accumulation of clinical experience.

## Introduction

Lung malignancy is one of the most common types of malignant tumors in terms of incidence rate (1, 2). Due to advancements in high-resolution computed tomography (HRCT), an increasing number of patients with multiple pulmonary nodules and multiple lung cancers (MLC) are being diagnosed. On the contrary, the radiological differentiation of lung malignancies can still be challenging at times, especially when accompanied with other benign pulmonary conditions (3). While lung cancer is highly prevalent, the co-occurrence of lung cancer with tuberculosis or pulmonary hamartoma is rare. To the best of our knowledge, cases of lung cancer combined with tuberculosis as well as pulmonary hamartoma have not been reported. Lung cancer combined with tuberculosis not only promotes the deterioration of lung cancer but also leads to the activation of tuberculosis (4, 5). It is reported that pulmonary hamartomas also have the potential for malignancy (6). In this paper, we present a recent case treated at the Second Hospital of Jilin University, involving a patient with primary lung adenocarcinoma combined with lung squamous cell carcinoma, pulmonary hamartoma, and pulmonary tuberculosis. Our aim is to provide a comprehensive analysis of this unique case and enhance the understanding of this particular clinical scenario.

## Case report

**Figure 1:**
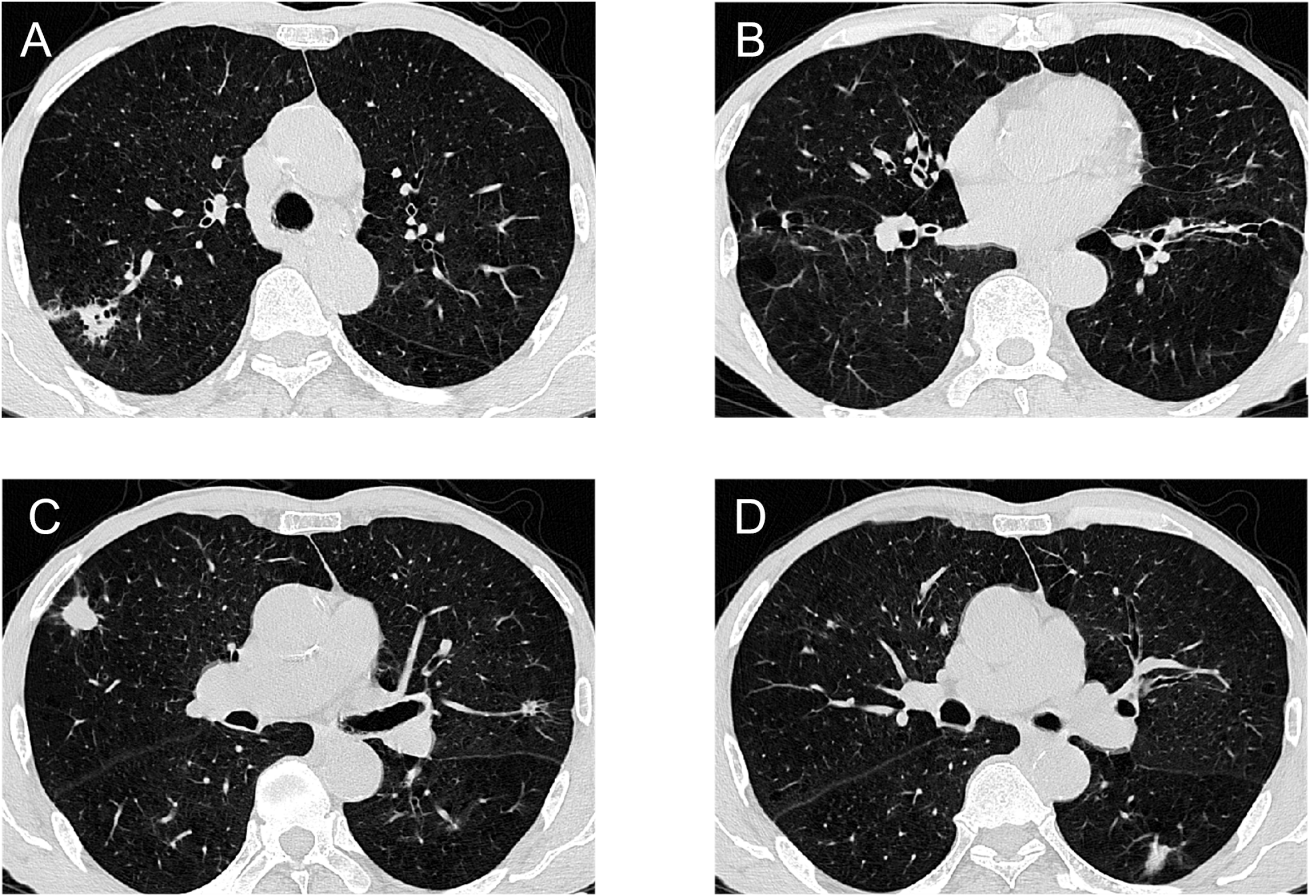
Chest CT results of the patient: **(A)**Right upper lung mass **(B)**. Right lower lung mass **(C)**Left upper lung mass and right upper lung mass **(D)**Left lower lung mass.

**Figure 2:**
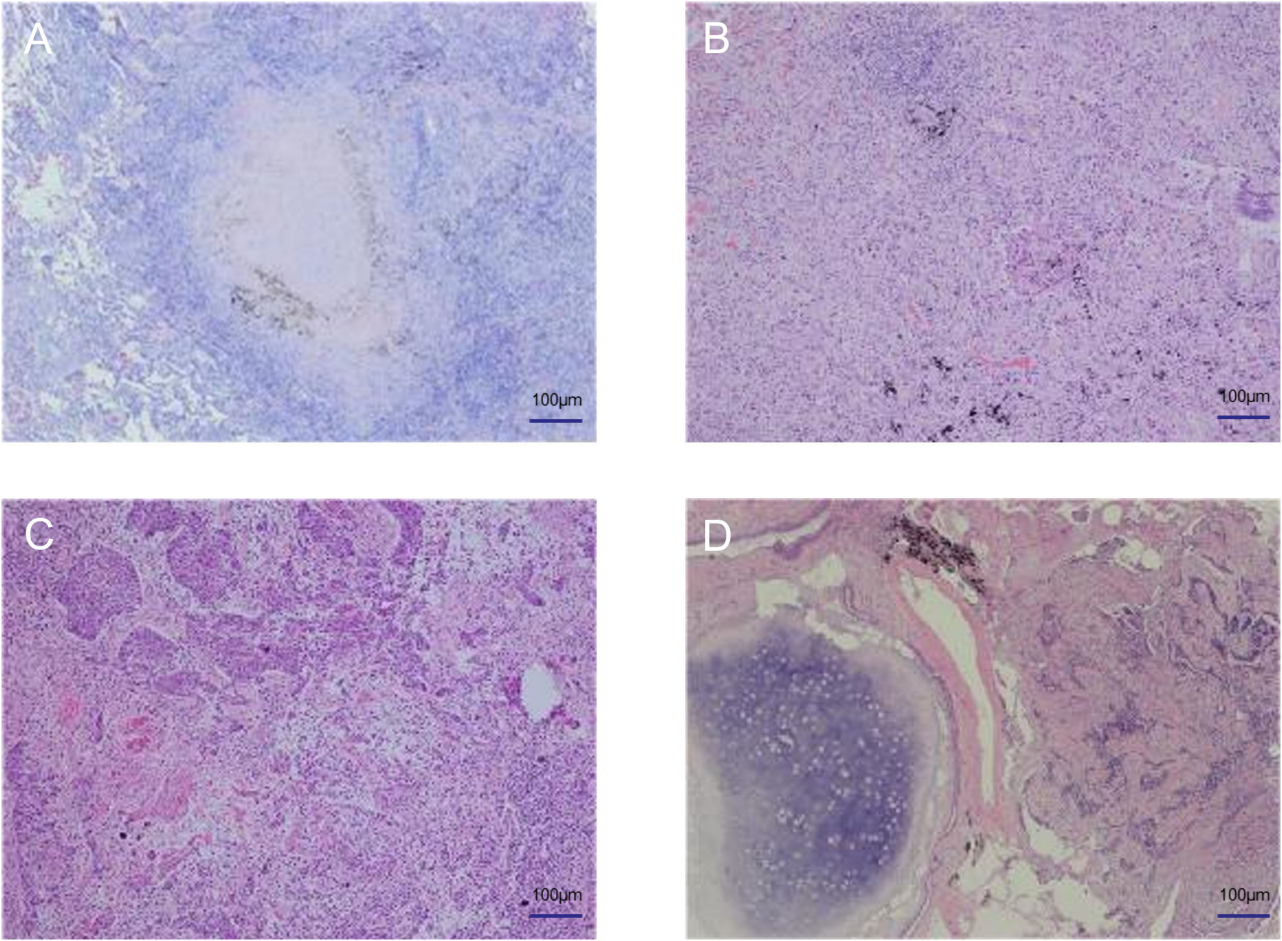
Pathological findings of the patient. **(A)** Tuberculosis **(B)** Adenocarcinoma **(C)** Squamous cell carcinoma **(D)** Hamartoma.

A 73-year-old male patient was found to have multiple lung nodules during a routine chest Computed Tomography (CT) scan conducted one month ago. Among these, the nodule in the anterior segment of the right upper lobe(RUL) measures 18.3mm, the nodule in the posterior segment of the RUL measures 18.6mm, the nodule in the dorsal segment of the right lower lobe(RLL) measures 20.1mm, the nodule in the posterior apical segment of the left upper lobe(LUL) measures 6mm, and the nodule in the dorsal segment of the left lower lobe (LLL) measures 16.2mm. The patient's Chest CT results are shown in **Figure 1**. To rule out lung nodules caused by inflammatory conditions, the patient underwent anti-inflammatory treatment. On reevaluation with positron emission tomography-CT(PET-CT)after 14 days, multiple abnormal density shadows were detected in both lungs, accompanied by increased metabolic activity, suggesting malignancy. Among these, the SUVmax values were 13.94 for the nodule in the anterior segment of the RUL, 8.17 for the nodule in the posterior segment of the RUL, 11.39 for the nodule in the RLL, 7.23 for the nodule in the LUL, and 5.3 for the nodule in the LLL. The laboratory test results showed no abnormalities in the blood routine, coagulation function, liver function, and kidney function. The results of the human immunodeficiency virus (HIV) antibody, hepatitis B 5 items, and hepatitis C antibody/syphilis antibody tests were all negative. The physical examination revealed a body temperature of 36.2°C, respiratory rate of 16 breaths per minute, pulse rate of 78 beats per minute, and blood pressure of 144/88 mmHg. The patient predominantly exhibited abdominal breathing with symmetrical respiratory movements. Clear breath sounds were heard in both lungs. The electrocardiogram showed sinus rhythm, indicating a generally normal cardiac activity. Thus, we decided to perform staged video-assisted thoracoscopic wedge resection of the lung lesions in order to achieve complete tumor removal. First, the patient underwent RUL wedge resection and RLL wedge resection in the left lateral decubitus position using video-assisted thoracoscopic surgery (VATS). Preoperatively, the patient’s blood gas analysis showed a pH of 7.41, pCO2 of 40mmHg, pO2 of 76mmHg, HCO3- of 25.4mmol/L, BE of 0.7mmol/L, and SaO2 of 95%. One week later, the patient underwent LUL wedge resection and LLL wedge resection in the right lateral decubitus position using VATS. Preoperatively, the patient’s blood gas analysis showed a pH of 7.49, pCO2 of 36mmHg, pO2 of 72mmHg, HCO3- of 27.4mmol/L, BE of 4.1mmol/L, and SaO2 of 96%. The intraoperative pathological diagnosis revealed squamous cell carcinoma for the nodule in the RUL(Nodule 1), chronic granulomatous inflammation suggestive of tuberculosis for the nodules in the RUL(Nodule 2), and LLL, invasive adenocarcinoma for the nodule in the RLL, and hamartoma for the nodule in the LUL. Postoperative pathological examination revealed the following findings: The nodule in the RUL (Nodule 1) is consistent with moderately to poorly differentiated squamous cell carcinoma, supported by immunohistochemical staining and morphological features. The tissue sample from the second nodule in the RUL exhibited extensive necrosis, surrounded by chronic granulomatous inflammation. The nodule in the RLL is diagnosed as invasive adenocarcinoma, primarily exhibiting a follicular pattern, with additional findings of an adherent growth pattern and a small amount of complex glandular structures. The patient's Pathological findings are shown in **Figure 2**. No definite vascular invasion was observed. The immunohistochemical staining results were as follows: CK (AE1/AE3) (+), TTF-1 (-), NapsinA (-), CK7 (+), CK5/6 (+), P40 (+), Ki67 (80% positivity), CD34 (-), CK8/18 (weak +), vimentin (focal +), INSM1 (-), BRG-1 (+),INI-1(+).Special staining results were as follows: PAS stain(-), antacid stain(-). Postoperatively, the patient underwent genetic testing, which did not detect any gene mutations associated with lung cancer. Furthermore, the patient was staged as pT1b, and no further treatment was deemed necessary. The patient has fully recovered and has been discharged from the hospital.

## Discussion

The widespread use of low-dose chest CT has led to an increased detection rate of early-stage malignant lung tumors. Nonetheless, even with the addition of PET-CT, chest CT still has certain limitations in diagnosing lung malignancies. Firstly, the radiological presentation of the lung malignancy in this case is atypical. The patient’s chest CT showed a relatively low proportion of solid components in the nodule of the right lung’s lower lobe, presenting as ground-glass opacity(GGO). In contrast, the pathology revealed invasive adenocarcinoma. This reminds us that even if CT indicates pure GGO, it may still represent invasive adenocarcinoma. According to the report by Yoon et al., a consolidation tumor ratio (CTR) exceeding 75% is an independent risk factor for lung adenocarcinoma (7). According to the report by Shimomura et al., volume-based consolidation tumor ratio (CTR) can serve as a useful predictive indicator for accurate clinical staging of stage I and II adenocarcinoma patients (8). Based on a retrospective study of 985 cases, Lin et al. concluded that lesions with a lower proportion of solid components, such as pure GGO nodules, had significantly more favorable histological features and prognosis compared to the group with part-solid nodule (PSN) (9). Nevertheless, reliance on such predictions should be cautious, as they may not universally apply, and treatment decisions based solely on radiological measurements of GGO tumors are inappropriate. Research has demonstrated that radiological measurements of lung adenocarcinoma, including consolidation-to-tumor ratio value, consolidation size, and tumor size, cannot accurately predict tumor invasion and prognosis. Accordingly, formulating treatment strategies based solely on radiological measurements of GGO tumors is inappropriate (10). Li et al. acknowledged that the CTR was an independent prognostic factor for the entire non-small cell lung cancer (NSCLC) population, but not for certain subtypes of NSCLC. Tumor size still holds significance for certain solid NSCLC cases (11). Hence, in clinical practice, even for pure ground-glass nodules (GGNs) detected on CT, it is important to consider multiple factors such as tumor diameter, serum tumor markers, maximum standardized uptake value on PET-CT, age, gender, smoking status, current extra-pulmonary cancer, air bronchogram and nodule size (p<0.05),circulating tumor cell,and others (12, 13). After comprehensive evaluation, an appropriate treatment plan can be chosen. Relying solely on imaging findings may introduce certain biases.

PET-CT has become a widely employed diagnostic tool for malignant lung tumors. Various studies have demonstrated the valuable role of PET-CT in disease identification, lesion localization, differentiation between benign and malignant nodules, and assessment of invasiveness and vascular involvement of lung lesions (14–17). Nonetheless, in this patient, increased uptake of FDG was observed on PET-CT in both the left upper lobe hamartoma and the left lower lobe tuberculosis. The correct diagnosis of hamartoma and lung tuberculosis was only established through surgical resection and pathological examination. This finding is noteworthy as hamartomas and lung tuberculosis can potentially present as false-positive results on PET-CT. As reported, the sensitivity and specificity of CT in distinguishing benign nodules from malignant nodules are approximately 80-90% and 50-60%, respectively (18). However, PET-CT scans may have limitations due to false-positive results in cases of infection or inflammation (19). This report describes a case where lung tuberculosis combined with a hamartoma was initially misdiagnosed as a malignant lung tumor. The presence of lung tuberculosis led to increased uptake of FDG, resulting in a false-positive result (20). Furthermore, there have been studies summarizing 19 cases of false-positive nodules on PET-CT scans, where high standard uptake values (SUV) above the threshold of 2.5 during PET-CT scanning were labeled as malignant tumors. This report indicates that tuberculosis, aspergilloma, bronchiolitis obliterans organizing pneumonia (BOOP), sarcoidosis, sequestration, anthracosis, and hamartoma can all exhibit high uptake on PET scans, leading to false-positive results (21). A study by Sathekge et al. demonstrated that there was no significant difference between the mean %DSUV of benign lesions 17.1%(SD 16.3%) and malignant lesions 19.4%(SD 23.7%). They concluded that PET-CT is unable to differentiate between malignant tumors and tuberculosis (22). Cicco et al. confirmed a series of PET-CT high-uptake pulmonary hamartomas through pathological examination. Their results revealed that approximately 20% of hamartomas exhibited uptake characteristics suggestive of malignant tumors (23). Goo et al. compared the uptake patterns of PET-CT scans in 10 cases of pulmonary tuberculosis nodules. Their findings demonstrated high FDG uptake (range: 9.3-7.21) near the primary lesion in four patients. Whereas, histopathological examination revealed chronic granulomatous inflammation with caseous necrosis or healed tuberculosis with fungal balls. Consequently, they concluded that when interpreting positive PET-CT results for differentiating between benign and malignant pulmonary masses, consideration should be given to the possibility of pulmonary tuberculosis (24). The above research results indicate that both pulmonary tuberculosis and hamartomas can indeed show increased FDG uptake. Accordingly, when encountering a positive PET-CT result, a comprehensive evaluation of the lesion’s size, morphology, and symptoms should be conducted to avoid inappropriate diagnosis and treatment resulting from false-positive findings. Simultaneously, preoperative pathological examination is crucial, as we can determine the benign or malignant nature of the lesion through fine needle aspiration pathology examination.

Thirdly, we should pay attention to the relationship between pulmonary tuberculosis and lung cancer. In the case of this patient, both pulmonary tuberculosis and lung cancer coexisted, prompting an exploration of the potential mutual influence between the two conditions. Firstly, pulmonary tuberculosis has been found to have a promoting effect on lung cancer. Sanchez et al. concluded that the potential risk of lung cancer was higher after an episode of tuberculosis (25). Preda et al. mentioned in their article that genomic modifications, chronic inflammation, and fibrosis resulting from tuberculosis may contribute to carcinogenesis. Therapies used for lung cancer, such as checkpoint inhibitors, may reactivate latent tuberculosis or exacerbate its progression. As a consequence, it is important to prioritize the treatment of concurrent pulmonary tuberculosis and lung cancer (26). Through meta-analysis, Abdeahad et al. concluded that active pulmonary tuberculosis increases the risk of lung cancer. Their findings indicate an association between active pulmonary tuberculosis and an elevated risk of adenocarcinoma, squamous cell carcinoma, and small cell lung cancer. Additionally, they concluded that the risk of developing lung cancer remains elevated even after recovery from pulmonary tuberculosis (4). On the other hand, Heuvers et al. argued that a history of pulmonary tuberculosis may serve as an important prognostic factor for lung cancer survival (27). The coexistence of pulmonary tuberculosis and lung cancer is relatively rare. The mechanism underlying this phenomenon may be attributed to several hypotheses, as follows. Firstly, old pulmonary tuberculosis lesions can cause persistent inflammation, leading to fibrosis and scar formation, which enhances the accumulation of carcinogenic substances in that area. The formation of pulmonary scars after repeated tuberculosis treatment favors tumor growth. This type of cancer is known as scar cancer (28). Additionally, the cell wall components of Mycobacterium tuberculosis induce the production of nitric oxide and reactive oxygen species, both of which contribute to the carcinogenic process (29). Conversely, lung cancer also plays a promoting role in the development of pulmonary tuberculosis. Research has shown that within one year of the diagnosis of respiratory or digestive tract cancer, tobacco-related cancer, or hematological malignancies, there is a significantly increased risk of developing tuberculosis. They believe that this may be related to reduced infection barriers, immune suppression, and shared risk factors, and they consider cancer as a clinical predictive indicator for increased active pulmonary tuberculosis cases (5). Hence, we can consider that there is a bidirectional relationship between lung cancer and pulmonary tuberculosis. When faced with cases involving the coexistence of lung cancer and pulmonary tuberculosis, apart from conventional surgical treatment, the management of tuberculosis and long-term postoperative follow-up are critical.

In addition, pulmonary hamartomas also have the potential to transform into cancer. Karasik et al., through a clinical study of 1976 patients with chondromatous hamartoma, concluded that the risk of chondromatous hamartoma developing into lung cancer is significantly higher compared to the general population (30). Lee et al. reported a case of pulmonary squamous cell carcinoma originating from a hamartoma. The pathological results of the patient revealed that the pulmonary hamartoma and squamous cell carcinoma were present within the same nodule. They concluded that pulmonary hamartomas can develop into squamous cell carcinoma (31). Ribet et al. also suggested that pulmonary hamartomas can develop into lung cancer, and they believe that smoking and environmental carcinogens play a crucial role in this transformation process (32). In summary, we have found that the coexistence of pulmonary hamartoma and malignant lung tumors is rare in clinical practice. Nonetheless, there is a possibility of malignant transformation in pulmonary hamartomas. Accordingly, even in histopathologically confirmed cases of pulmonary hamartoma, follow-up examinations should be conducted to prevent potential malignant transformation.

## Conclusions

We present a case involving the simultaneous occurrence of pulmonary adenocarcinoma, squamous cell carcinoma, hamartoma, and pulmonary tuberculosis. The patient’s PET-CT images of the lesions showed increased FDG uptake. Nevertheless, as previously discussed, PET-CT has limited capability in distinguishing between lung cancer, hamartoma, and pulmonary tuberculosis. As a consequence, these nodules were identified with a higher likelihood of malignancy. Remarkably, tuberculosis lesions were observed in both lungs of the patient. We hypothesize that the chronic inflammation and fibrosis resulting from recurrent pulmonary tuberculosis infections may have created an environment favorable for the development of cancer, ultimately leading to its occurrence.

## Data availability statement

The original contributions presented in the study are included in the article/supplementary material. Further inquiries can be directed to the corresponding authors.

## Ethics statement

The studies involving humans were approved by Ethics Committee of the Second Hospital of Jilin University. The studies were conducted in accordance with the local legislation and institutional requirements. The participants provided their written informed consent to participate in this study. Written informed consent was obtained from the individual(s) for the publication of any potentially identifiable images or data included in this article.

## Author contributions

JK: Conceptualization, Writing – original draft. MW: Investigation, Writing – original draft. PH: Validation, Writing – review & editing. BW: Supervision, Validation, Writing – review & editing.
